# Garlic Increases Antioxidant Levels in Diabetic and Hypertensive Rats Determined by a Modified Peroxidase Method

**DOI:** 10.1093/ecam/nep011

**Published:** 2010-10-20

**Authors:** Hana Drobiova, Martha Thomson, Khaled Al-Qattan, Riitta Peltonen-Shalaby, Zainab Al-Amin, Muslim Ali

**Affiliations:** Department of Biological Sciences, Faculty of Science, Kuwait University, P.O. Box 5969, 13060-Safat, Kuwait

## Abstract

Oxidative damage by free radicals has been implicated in the pathogenesis of vascular disease in diabetes and hypertension. In the present study, the total antioxidant status in diabetic and hypertensive rats before and after treatment with garlic (*Allium sativum*) was determined. The total serum antioxidants were measured by a modified method reported earlier by Miller and coworkers. The reproducibility of the assay was confirmed by determining standard curves for the known antioxidants: trolox (a stable analog of vitamin E), glutathione and vitamin C with interassay correlation coefficients (*R^2^,* *n* = 10 in triplicate) of 0.9984, 0.9768 and 0.987, respectively, confirming the reliability and reproducibility of the assay. This assay was then used to determine total serum antioxidant levels of streptozotocin-induced diabetic rats and two-kidney one-clip hypertensive rats both before and after 3 weeks of treatment with an aqueous extract of garlic (500 mg/kg IP daily). The serum antioxidant levels of rats after 3 weeks of treatment were significantly higher (*P* < .001) than the pretreatment levels in both diabetic and hypertensive rats. The increased serum antioxidant levels were paralleled by a decrease in serum glucose in the garlic-treated diabetic rats and lowered systolic blood pressure in the garlic-treated hypertensive rats. We conclude from our study that (i) total antioxidants can be measured by a simple, reproducible, reliable assay and (ii) the total antioxidant status can be significantly improved by treatment with garlic.

## 1. Introduction

Oxidative stress, an excessive production of reactive oxygen species (ROS) above the body's antioxidant capacity, has been implicated in the development of many pathophysiological conditions including hypertension, diabetes, atherosclerosis and cancer, as well as the process of aging [1–6]. ROS are normal products of cellular metabolism which are usually removed by endogenous antioxidants [7]. However, it has become increasingly clear that overproduction of ROS can lead to a damaging cycle of lipid peroxidation, depletion of natural antioxidants such as glutathione, perturbation of nitric oxide production and disruption of normal cellular metabolism [8]. These changes have been shown to cause damage to cell membranes and in particular can cause endothelial dysfunction [9].

Due to the importance of changes in the oxidant to antioxidant ratio in relation to the development of pathological conditions, the measure of antioxidant capacity of serum has increasingly become used as an index of the antioxidant status of the system. Markers of oxidative stress that are commonly measured include: endogenous antioxidants such as glutathione, cysteine and antioxidant vitamins; antioxidant enzymes such as glutathione peroxidase, myeloperoxidase and superoxide dismutase; F_2_-isoprostanes and malondialdenyde as products of lipid peroxidation; and end-products of protein oxidation such as nitrotyrosine [8, 10]. In addition, a number of *in vitro* spectrophotometric assays have been developed to assess antioxidant capacity including the quantitation of Trolox equivalent antioxidant capacity [8, 11]. However, many of these assays are laborious, involve the use of difficult and unclear methodologies and are expensive. In a recent review, Dalle-Donne and coworkers have discussed the measurement and reliability of various biomarkers in human disease [10].

Recent reports have suggested that diabetic complications and hypertension have a common etiology involving oxidative stress and endothelial damage [9, 12, 13]. In fact, the earliest pathological changes in diabetic complications have been reported to be hemodynamic in nature [14–16]. Thus, the assessment of oxidative stress in both diabetes and hypertension has become crucial as an index of pathology of these diseases. In fact, many reports have emphasized the common link between ROS levels and severity of these conditions [17, 18]. In addition, antioxidants have been shown to modulate oxidative stress-induced changes in hypertension, atherosclerosis and diabetes [19, 20].

Due to the increasing worldwide prevalence and financial burden of chronic diseases such as diabetes and hypertension, it has become increasingly important to find pharmacological remedies to alleviate the symptoms and complications of these conditions. In particular the use of natural remedies such as garlic has become popular as both preventative and treatment alternatives. Garlic has been extensively studied and has been shown to have a number of medicinal properties including antithrombotic [21–26], antibiotic [27–29], hypolipidemic [30, 31], hypocholesteremic [30, 32], hypoglycemic [33–39] and hypotensive [30, 40] activities. Various garlic preparations and components have also been shown to have antioxidant activity including the ability to lower ROS *in vivo* [31, 41, 42].

In recent years, we have been studying the beneficial effects of aqueous extracts of garlic in the treatment of chronic diseases such as diabetes and hypertension [21, 23–25, 30, 43, 44]. As part of this work, we found it necessary to develop a method for measurement of oxidative stress. Measurement of Trolox equivalent antioxidant capacity units (TEAC) was developed by Miller, Rice-Evans and coworkers as a method for measurement of antioxidant capacity [45–47]. This is an electron-transfer method that exploits the peroxidase activity of methemoglobin combined with its interaction with a phenothiazine compound [azinobis-(3-ethylbenzothiazoline-6-sulfonic acid), ABTS] to form a radical cation intermediate (ABTS^•+^). The concentration of antioxidants giving the same percentage change of absorbance of ABTS^•+^ as that of 1 mM Trolox is regarded as a TEAC [48]. However, description of this method lacked critical details necessary to its routine use. Therefore, in the present study, the assay was modified for routine measurement of antioxidant capacity of serum and was applied to the assessment of antioxidant levels in diabetic and hypertensive rat models treated with garlic.

## 2. Materials and Methods

### 2.1. Materials

Streptozotocin, myoglobin (equine) and 2,2′-azinobis-(3-ethylbenzothiazoline-6-sulphonic acid) diammonium salt (ABTS) were purchased from Sigma Chemical Co. (St Louis, MO, USA). Trolox (6-hydroxy-2,5,7,8-tetramethylchronam-2-carboxylic acid) was purchased from Aldrich Chemical Co. (Milwaukee, WI, USA). Vitamin C, reduced glutathione and hydrogen peroxide were obtained from Fluka (Buchs, Switzerland). Glucose oxidase kits were obtained from Waco Pure Chemicals Co., Ltd (Osaka, Japan). All other reagents were of analytical grade.

### 2.2. Antioxidant Assay

Total antioxidant levels were measured by a modified Rice-Evans method [45–47] which is based on the inhibition by antioxidants of the absorbance of the radical cation ABTS^•+^. The spectrophotometric method derives from the observation that when ABTS is incubated with a peroxidase (metmyoglobin) and hydrogen peroxide, the relatively long-lived radical cation, ABTS^•+^, is formed. ABTS^•+^ has absorption maxima at 650, 734 and 820 nm. In the presence of antioxidants, the absorbance (at 734 nm) of this radical cation is quenched to an extent and on a time scale dependent on the antioxidant capacity of the material under investigation.

Metmyoglobin (MetMb) was prepared by mixing myoglobin with an oxidizing agent [K_3_Fe(CN_6_)] and then re-purified on a G-25 column before use as described by Miller and Rice-Evans [47]. The concentration of myoglobin in the column eluate was calculated using the extinction coefficients of met-, oxy- and ferryl-myoglobin at 490, 560 and 580 nm using the following equation:

$$[\text{MetMb}](\mu \text{M})=146A_{490}-108A_{560}+2.1A_{580}$$.

Purified myoglobin was stored in small aliquots at −20°C until use.

The current modification of the assay included changes in the specificity of timing of the assay including a 3 min preincubation that are critical to the reproducibility of the assay (Table 1). Three known antioxidants, vitamin C, trolox (a stable analog of vitamin E) and glutathione, were used to assess the reproducibility of the assay. The results are calculated and expressed as TEAC.

### 2.3. Preparation of Garlic Extract

Aqueous garlic extract was prepared from locally available garlic cloves. The garlic cloves were peeled on crushed ice and 50 g of garlic was cut into small pieces and homogenized in 75 mL of cold, sterile 0.9% NaCl in the presence of some crushed ice. The homogenization was carried out in a blender at high speed using six 2-min bursts for a total of 12 min. The homogenized mixture was filtered three times through cheesecloth. The filtrate was centrifuged at 2000 RCF for 10 min and the clear supernatant was made up to 100 mL with normal saline. The concentration of this garlic preparation was considered to be 500 mg/mL on the basis of weight of the starting material (50 g/100 mL).

### 2.4. Diabetes Induction and Sample Collection

Male Sprague-Dawley rats weighing 250–280 g and maintained on a normal diet and filtered tap water *ad libitum* were used in the study. All animal manipulations were conducted according to the ethical guidelines outlined in the Guide for Care and Use of Laboratory Animals [49]. For baseline data, blood was drawn from all animals by cardiac puncture under ether anesthesia and allowed to clot. Immediately, the clotted blood was centrifuged at 2000 RCF for 30 min. The serum was separated and stored at –80°C for later analysis.

The animals were randomly divided into a normal group (eight rats) and a streptozotocin (STZ)-treated group (20 rats). The STZ-treated rats were injected with 60 mg steptozotocin/kg body weight intraperitoneally (IP) in a volume of 0.5 mL saline according to the method of Axler [50] following an overnight fast. After a period of 3 days, blood was drawn from the STZ-treated animals by cardiac puncture, and serum was prepared and stored for later analysis as described above. Serum glucose levels were determined immediately and the STZ-treated rats determined to be diabetic due to a high serum glucose level (>350 mg/dL) were randomly divided into two groups containing eight animals each: Group 1, the diabetic control group, was injected IP daily with saline for the treatment period; and Group 2, the garlic-treated group, was injected IP daily with 500 mg/kg of the garlic extract.

After a period of 3 weeks, the rats were sacrificed under sodium pentobarbitone anaesthesia according to the guidelines for euthanasia in the Guide for Care and Use of Laboratory Animals [49]. Before sacrifice, blood was collected, processed and stored at −80°C for analysis within 1 week as described above.

### 2.5. Induction of Hypertension and Assessment

Ten male Sprague-Dawley rats with initial body weight of 50 g were operated on to induce a two-kidney, one-clip (2K-1C) model of hypertension as described earlier [51]. After 1 week, hypertension was confirmed by blood pressure measurement using the tail-cuff method. The hypertensive rats were then divided into two groups: five of the rats were treated daily with 500 mg/kg garlic IP for a period of 3 weeks and the remaining five rats served as control and were given saline IP for 3 weeks. Blood pressure was also measured at the end of the treatment period by the tail-cuff method. Blood was drawn by cardiac puncture before and after 3 weeks of treatment as described above and total antioxidant levels were determined.

### 2.6. Assays

Serum glucose was quantitated spectrophotometrically by the glucose oxidase method using kits from Waco, (Japan). TEAC levels (mM) were determined as described above. Systolic blood pressure was measured using the tail-cuff technique (Harvard Apparatus, England).

### 2.7. Statistical Analysis

The experimental data are expressed as mean ± SEM. Statistical analysis was performed using Minitab Statistical Software (Version 7, State College, PA, USA). Data was analyzed using the two-sample *T*-test and a level of *P* < .05 is considered to be significant.

## 3. Results

### 3.1. Assessment of the Assay

**Figure 1:**
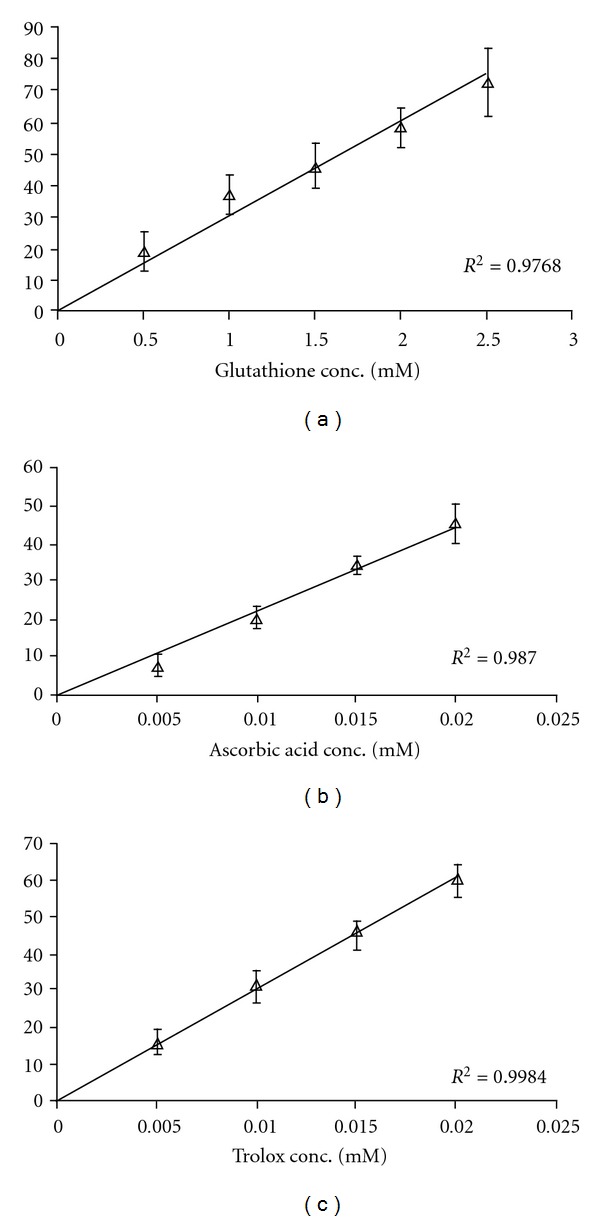
Standard curves for inhibition of ABTS^+•^ absorbance at 734 nm with (a) glutathione, (b) ascorbic acid (vitamin C) and (c) trolox. Glutathione concentrations were between 0.5 and 2.5 mM. Ascorbic acid concentrations were between 0.005 and 0.02 mM. Trolox concentrations were between 0.005 and 0.02 mM. All assays were done in triplicate.

The reproducibility of the antioxidant assay was assessed by determination of the inhibitory effects of three known antioxidants, vitamin C, glutathione and trolox (a stable analog of vitamin E). The standard curve for each antioxidant was performed 8–10 times in triplicate. The standard curves in Figures 1(a)–1(c) indicate that for all three known antioxidants good reproducibility within the concentration ranges was obtained using the assay. The inter-assay correlation coefficients (*R^2^*) were 0.9984, 0.9768 and 0.987 for trolox, glutathione and vitamin C, respectively, confirming the reliability and reproducibility of the assay.

### 3.2. Effects in Diabetic Rats

**Figure 2:**
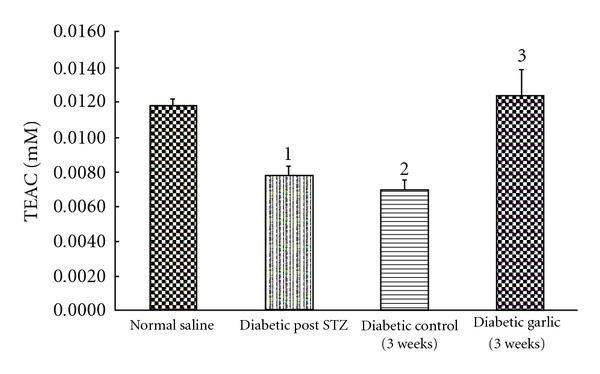
Antioxidant levels in diabetic rats. Normal, untreated (control) diabetic and garlic-treated diabetic (diabetic garlic) rats were assessed for antioxidant status (TEAC) 1 week after STZ-injection (Post-STZ) and 3 weeks after treatment. (1) Total antioxidants in normal rats are significantly hgher than in diabetic rats 3 days after induction of diabetes with STZ (*P* < .05). (2) Total antioxidants in normal rats are significantly higher than in diabetic control rats after 3 weeks (*P* < .05). (3) Total antioxidants in diabetic control rats are significantly lower than in garlic-treated diabetic rats after 3 weeks of garlic treatment (*P* < .05).

Induction of a diabetic state with STZ led to marked hyperglycemia within 3 days of injection (Table 1). This hyperglycemic state was accompanied by significantly decreased antioxidant levels (about 40% reduction) both 3 days and 3 weeks after STZ-injection (Figure 2). In contrast, after 3 weeks, the diabetic rats given 500 mg/kg garlic daily recovered antioxidant activity reaching levels in excess of those observed in normal rats. This increase in serum antioxidant levels was paralleled by a decrease in serum glucose levels as the garlic-treated diabetic rats returned to an euglycemic state (Table 2).

### 3.3. Effects in Hypertensive Rats

**Figure 3:**
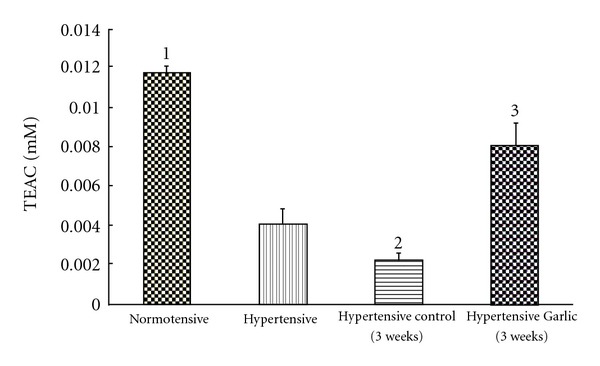
Antioxidant levels in hypertensive rats. Normal, untreated (control) hypertensive and garlic-treated hypertensive rats were assessed for antioxidant status (TEAC) before and 3 weeks after treatment. (1) Total antioxidants in normotensive rats are significantly higher than in hypertensive rats (*P* < .05). (2) Total antioxidants in hypertensive rats at time zero are significantly higher than hypertensive rats after 3 weeks (*P* < .05). (3) Total antioxidants in untreated hypertensive rats are significantly lower than garlic-treated hypertensive rats after 3 weeks of garlic treatment (*P* < .05).

As shown in Figure 3, 2K-1C hypertensive rats (systolic BP = 200 ± 17 mm) exhibited significantly decreased total antioxidant levels compared to normotensive rats (about 66% reduction). After 3 weeks of garlic treatment, the antioxidant levels in the hypertensive rats increased to about two-third of the normal levels, while the antioxidant levels in the hypertensive control rats continued to decrease (about 20% of the normotensive level) during the 3-week experimental period. This increase in antioxidant levels occurred simultaneously with a 50% decrease in blood pressure (to 100 ± 22 mm systolic) in the 2K-1C hypertensive rats.

## 4. Discussion

During the last decade, it has become increasingly evident that many chronic diseases are accompanied by increased levels of oxidative stress exacerbated by decreased antioxidant levels [10, 17, 52–54]. These observations have precipitated much interest in study of the correlations between oxidative stress, antioxidant potential and development of chronic diseases in both humans and animal models. Of particular interest are the correlations between oxidative stress and development of diabetes and hypertension [4, 5, 9, 55–57].

With the surge in research in this area, the development of simple, reliable and consistent methods for quantitation of oxidative stress has become imperative. In particular, assays for serum antioxidant levels have been developed including measurement of total peroxy radical trapping as described by Wayner et al. [58] and Rice-Evans and Miller [45–47]. Whereas the original peroxy radical trapping parameter assay of Wayner and coworkers [57] required an oxygen electrode endpoint, Rice-Evans and Miller developed this assay to use a spectrophotometric endpoint [45]. As described above, this assay involves utilization of the peroxidase activity of methemoglobin, generation of the radical cation, ABTS^•+^, and quantitation of the quenching of this radical cation by antioxidants. Results are expressed in Trolox equivalent antioxidant capacity using the vitamin E analog, Trolox, as the reference antioxidant.

In the present study, we have modified the Rice-Evans and Miller assay [46, 47] to include more precise timing parameters including a 3-min pre-incubation, a 6-min reaction time followed by absorbance measurement exactly 15 s after stopping the inhibition reaction. These timing parameters have allowed the assay to be conducted with the use of a 30°C water bath and a basic spectrophotometer. The Rice-Evans and Miller manual assay [46] required the use of a temperature-controlled cuvette holder for continuous measurement of absorbance. Thus, in the current modification, the assay involves pre-incubation of all reagents except H_2_O_2_ for 3 min at 30°C, addition of H_2_O_2_ at 15 s intervals followed by 6 min incubation at 30°C. The absorbance was then read at exactly 15 s intervals such that all assays were incubated for exactly 6 min. These timing parameters allowed six assays to be carried out simultaneously. The 6 min reaction time is in agreement with that of van den Berg et al. [59] and Re and coworkers [60], who also modified the assay to eliminate the use of methemoglobin. In our hands, the modifications described have allowed the routine use of this assay for assessment of serum antioxidant levels. Standard curves for three known antioxidants (gluathione, vitamin C and Trolox) indicate that the assay is reproducible and stable (Figures 1(a)–1(c)).

There has been considerable focus in recent years on the connection between oxidative stress and endothelial dysfunction leading to the development of chronic conditions such as diabetes and hypertension [5, 9, 13, 56, 61]. Endothelial dysfunction is correlated with the development of coronary heart disease and peripheral vascular resistance which are the leading causes of morbidity and mortality in diabetes mellitus [18, 62]. It has become increasingly evident that the development of hyperglycemia followed by oxidative stress is the underlying cause of the chronic complications of diabetes. Therefore, the prevention or alleviation of oxidative stress in diabetes should positively benefit the diabetic patient.

Similarly, the development of hypertension has been linked to impairment in the antioxidant defense system [5, 56]. Similar to diabetes, hypertension, in particular renovascular hypertension, has been characterized by increased oxidative stress and endothelial dysfunction [63]. Therefore, numerous studies have focused on the correlation between serum antioxidant levels and hypertension as well as the alleviation of this chronic condition through increased consumption of antioxidants and antioxidant-rich foods [64, 65].

Our results show that treatment of STZ-induced diabetic rats with a raw garlic extract alleviates both hyperglycemia and oxidative stress in these animals. These observations are in agreement with those of Anwar and Meki [31] who treated STZ-induced diabetic rats with garlic oil. In addition, recently Zalejska-Fiolka and coworkers [66] reported that garlic-fed rats also fed oxidized oil had improved antioxidant status, less lipid peroxidation and fewer atherosclerotic changes than in oxidized oil fed controls. Similarly, Souza and coworkers [67] have reported that *N*-acetylcysteine (NAC), a water-soluble compound found in garlic extracts, improved high-sucrose diet-induced obesity in rats. NAC treatment resulted in improved glucose tolerance and lipid profile, as well as decreased LDL-oxidation and serum oxidative stress. Therefore, several studies have suggested that garlic most likely exacerbates diabetes in animal models through improved antioxidant status.

Therefore, we have shown the applicability of the TEAC assay to the assessment of antioxidant status of diabetic rats. It should be noted that the method described here measures only water-soluble antioxidants. Therefore, the results reported here do not include fat-soluble antioxidant levels which also may be affected by garlic treatment.

In previous studies, we have reported that the 2K-1C rat model is characterized by the development of renal hypertension accompanied by increased expression of two isoforms of the Na/H exchanger (NHE-1 and-3) in both kidneys and increased serum levels of PGE_2_ and TXB_2_ [43, 68]. Treatment of these rats with an aqueous extract of garlic lowered blood pressure and NHE-1 expression in the unclipped kidney [43]. In the present study, we have observed that the development of renal hypertension in the 2K-1C rat is accompanied by decreased levels of serum antioxidants. The increased blood pressure and oxidative stress were alleviated by administration of an aqueous extract of garlic. These results are in agreement with our earlier observation that the hypotensive effect of garlic is partially mediated through the nitric oxide pathway in the 2K-1C rat model confirming the usefulness of garlic in decreasing oxidative stress and hypertension [44].

## 5. Summary

From this study, the following conclusions can be stated. A reliable and reproducible assay for determination of total antioxidant levels in serum has been developed and applied. Treatment of diabetic rats with garlic resulted in significantly increased antioxidant and lowered glucose levels compared to untreated diabetic animals. Treatment of 2K-1C hypertensive rats with garlic increased the total level of antioxidants to about two-third of the normal level. Garlic-treated 2K-1C hypertensive rats had significantly higher total antioxidant levels and lower systolic blood pressure than untreated 2K-1C hypertensive rats.

## Figures and Tables

**Table 1:** Protocol for the antioxidant assay.

Solution	Blank^a^ (*μ*L)	Sample (*μ*L)
PBS (5 mM, pH 7.4, 138 mM NaCl)	1000 – (105 + *x*)	1000 – (115 + *x*)
MetMb (as determined above)	*x* ^b^	*x*
Sample (serum or antioxidant)	—	10
ABTS (5 mM)	30	30
Equilibrate for 3 min at 30°C
H_2_O_2_ (10 mM)	75	75
Incubate for 6 min at 30°C
Read absorbance at 734 nm after exactly 15 s of incubation period completion^c^

^a^ Read against PBS buffer.

^b^ MeMb concentration is determined experimentally and the required volume (*x*) is added to obtain 2.5 *μ*M final concentration in the assay. The volume of PBS buffer is adjusted accordingly.

^c^ Only six samples can be run at a time to fit the timing protocol.

**Table 2:** Serum glucose concentration in different animal groups.

	Initial serum glucose (mg/dL)	Final serum glucose (mg/dL)
Normal^a^ (saline treated)	—	129 ± 9
Diabetic control^a^ (Saline treated)	400 ± 9	431 ± 13^b^
Garlic-treated diabetic^a^ (500 mg/kg garlic)	400 ± 9^c^	237 ± 8^c^

^a^ *n* = 10 for all groups.

^b^ Significally different compared to normal (*P* < .05).

^c^ Significally different compared to initial and final diabetic controls (*P* < .001).
